# Testing the link between genome size and growth rate in maize

**DOI:** 10.7717/peerj.2408

**Published:** 2016-09-07

**Authors:** Maud I. Tenaillon, Domenica Manicacci, Stéphane D. Nicolas, Francois Tardieu, Claude Welcker

**Affiliations:** 1 Génétique Quantitative et Evolution—Le Moulon, INRA—Université Paris-Sud—CNRS—AgroParisTech, Université Paris-Saclay, Gif-sur-Yvette, France; 2 Ecophysiologie des Plantes sous Stress Environnementaux, INRA, Montpellier, France

**Keywords:** Adaptation, Leaf elongation rate, Zea mays, DNA content, Breeding

## Abstract

Little is known about the factors driving within species Genome Size (GS) variation. GS may be shaped indirectly by natural selection on development and adaptative traits. Because GS variation is particularly pronounced in maize, we have sampled 83 maize inbred lines from three well described genetic groups adapted to contrasted climate conditions: inbreds of tropical origin, Flint inbreds grown in temperate climates, and Dent inbreds distributed in the Corn Belt. As a proxy for growth rate, we measured the Leaf Elongation Rate maximum during nighttime (LER_max_) as well as GS in all inbred lines. In addition we combined available and new nucleotide polymorphism data at 29,090 sites to characterize the genetic structure of our panel. We found significant variation for both LER_max_ and GS among groups defined by our genetic structuring. Tropicals displayed larger GS than Flints while Dents exhibited intermediate values. LER_max_ followed the opposite trend with greater growth rate in Flints than in Tropicals. In other words, LER_max_ and GS exhibited a significantly negative correlation (*r* = − 0.27). However, this correlation was driven by among-group variation rather than within-group variation—it was no longer significant after controlling for structure and kinship among inbreds. Our results indicate that selection on GS may have accompanied ancient maize diffusion from its center of origin, with large DNA content excluded from temperate areas. Whether GS has been targeted by more intense selection during modern breeding within groups remains an open question.

## Introduction

It is well established that Genome Size (GS) varies greatly among species, and that much of this variation is caused by repeated sequences (Muñoz Diez et al., 2012; Grover & Wendel, 2010). There is still, however, a surprising dearth of studies assessing within-species variation. Among plant populations, several investigations have reported GS stability (Ellul et al., 2002; Moscone et al., 2003) while there are a handful of well-documented examples of substantial GS variation (reviewed in Smarda & Bures, 2010). The extent of within-species GS variation as measured by the coefficient of variation ranges from less than 1% in *Hordeum lechleri* (Jakob, Meister & Blattner, 2004), around 2% in *Arabidopsis thaliana* (Long et al., 2013), 3.4% in *Camellia sinensis* (Huang et al., 2013) and in *Festuca pallens* (Smarda, Bures & Horova, 2007), and up to 6% in maize (*Zea mays* ssp. *mays*) and its closest wild relatives (ssp. *parviglumis* and *mexicana*), the teosintes (Muñoz Diez et al., 2013).

The factors driving GS variation remain a largely controversial issue. Several competing models have been proposed to explain among-species variations in GS. Interestingly, at least two of these models involve population genetic processes that may drive GS variation within species among populations, and ultimately preside over among-species GS variation (Agren & Wright, 2011; Petrov, 2001). The “mutational hazard” hypothesis (Lynch et al., 2011) posits that selection to maintain a constant per-genome mutation rate indirectly impacts GS. Providing that selection overcomes drift, the per base-pair-per-generation mutation rate correlates negatively with GS (Sung et al., 2012). Under this model, one expects within-species GS variation to be driven by differences in effective population size that condition the efficiency of natural selection against genome expansion. An alternative hypothesis asserts that positive natural selection may indirectly influence GS variation through developmental or adaptive phenotypes (Knight & Beaulieu, 2008). In plants, the latter hypothesis has been sustained by a handful of empirical studies demonstrating that GS correlates negatively with development traits such as seedling (Mowforth & Grime, 1989), root meristem growth rate (Gruner et al., 2010), and cell cycle length (Francis, Davies & Barlow, 2008). Small genomes indeed presumably facilitate faster cell division and therefore a higher growth rate (Knight, Molinari & Petrov, 2005; Rayburn, Dudley & Biradar, 1994).

Improving our understanding of intra-species genome dynamics is essential for elucidating the diversification of GS among related species. Maize is an attractive model to test whether GS is fine-tuned by positive natural selection. Not only does it display the largest within-species GS variation in plants and an exceptional genome fluidity (Chia et al., 2012), but is also characterized by a large effective population size—with estimates ranging from 33,000 (Vigouroux et al., 2002) to ∼600,000 (Gossmann et al., 2010) and 993,000 individuals (Beissinger et al., 2016), and a worldwide distribution with contrasted growing conditions. Actually, maize has a long-lasting history of research on GS variation (for a review, see Knight, Molinari & Petrov, 2005). The most recent and extensive report on this question in maize landrace populations (Muñoz Diez et al., 2013) has drawn several important conclusions: (1) GS varies primarily among landraces and within-landrace variation is limited; (2) geographical coordinates (altitude, longitude, latitude) are accurate predictors of GS; (3) GS correlates negatively with altitude. These results corroborate significant GS difference between temperate and tropical inbred lines in a sample of 17 improved inbred lines as reported by Chia et al. (2012).

Altogether, these findings suggest that environmental-driven selection on life cycle length and growth rate could indirectly affect GS. To further validate this hypothesis, we measured GS and leaf elongation rate in 83 improved maize inbred lines of various origins in the purpose of establishing a link between GS and growth rate.

## Materials and Methods

We have sampled 83 maize inbred lines (inbreds) from the INRA Centre de Ressources Biologiques (Saint Martin de Hinx, France) and from the Maize gene bank at CIMMYT in Mexico (Table 1). In order to maximize GS and LER_max_ variation, we sampled inbred lines from three of the genetic groups previously defined by Camus-Kulandaivelu et al. (2006): tropical inbreds (Tropicals) characterized by a long life-cycle from sowing to flowering, flint inbreds (Flints) grown in temperate climates with a short life-cycle, and Dent inbreds (Dents) distributed in the Corn Belt with an intermediate life-cycle. Our panel encompassed 50 Tropicals, 18 Flints and 15 Dents.

**Table 1:** List of inbred lines with measures of Genome Size (GS), LERmax (LER) and membership at *K* = 2 (Group 1, 2) and *K* = 3 (Group 1, 2, 3).

Inbred line	GS (pg)		LER (mm/h)		K2_G1	K2_G2	K3_G1	K3_G2	K3_G3	K3_group
CH10	5.05	(0.026)	6.77	–	1.000	0.000	1.000	0.000	0.000	Flint
EP1	5.17	(0.129)	4.54	(0.149)	0.928	0.072	0.940	0.000	0.060	Flint
F39	5.31	(0.070)	6.25	(0.610)	0.879	0.121	0.894	0.000	0.106	Flint
F471	5.26	(0.101)	5.66	(0.113)	0.867	0.133	0.905	0.000	0.095	Flint
FC16	5.27	(0.027)	6.83	–	0.670	0.330	0.675	0.000	0.325	Flint
FC209	5.05	(0.090)	6.21	(0.047)	1.000	0.000	1.000	0.000	0.000	Flint
FC24	5.41	(0.093)	5.99	(0.045)	1.000	0.000	1.000	0.000	0.000	Flint
FV2	5.20	(0.069)	5.40	(0.251)	1.000	0.000	1.000	0.000	0.000	Flint
FV65	5.21	(0.020)	6.65	–	0.868	0.132	0.876	0.000	0.124	Flint
FV7	5.24	(0.055)	6.30	(0.514)	1.000	0.000	1.000	0.000	0.000	Flint
FV71	5.10	(0.020)	5.14	(0.129)	0.923	0.077	0.976	0.000	0.024	Flint
FV75	5.11	(0.041)	5.86	(0.575)	1.000	0.000	1.000	0.000	0.000	Flint
FV76	5.27	(0.089)	5.21	–	0.821	0.179	0.840	0.000	0.160	Flint
ND30	5.04	(0.047)	6.94	–	1.000	0.000	1.000	0.000	0.000	Flint
NY302	4.96	(0.057)	5.23	(0.269)	1.000	0.000	0.796	0.204	0.000	Flint
PB40R	5.28	(0.089)	5.04	(0.046)	0.770	0.230	0.725	0.087	0.187	Flint
W85	5.19	(0.045)	5.24	(0.397)	1.000	0.000	1.000	0.000	0.000	Flint
YUBR05	5.21	(0.073)	4.80	–	0.724	0.276	0.542	0.458	0.000	Flint
B73	5.21	(0.055)	5.42	(0.369)	0.490	0.510	0.000	1.000	0.000	Dent
CI1872U	5.26	(0.054)	4.73	(1.046)	0.305	0.695	0.000	0.729	0.271	Dent
EA1433	5.24	(0.052)	4.21	(0.519)	0.416	0.584	0.206	0.420	0.373	Dent
FC1852	5.33	(0.054)	6.12	(0.249)	0.494	0.506	0.000	1.000	0.000	Dent
FV252	5.23	(0.199)	4.80	(0.108)	0.449	0.551	0.000	1.000	0.000	Dent
K64R	5.24	(0.115)	5.68	(0.249)	0.313	0.687	0.052	0.538	0.410	Dent
KY21	5.20	(0.045)	5.56	(0.885)	0.416	0.584	0.000	1.000	0.000	Dent
LAN496	5.17	(0.050)	5.97	(0.009)	0.476	0.524	0.076	0.924	0.000	Dent
MBS847	5.17	(0.008)	4.45	(0.519)	0.437	0.563	0.000	1.000	0.000	Dent
MO17	5.16	(0.010)	4.78	(0.107)	0.448	0.552	0.000	1.000	0.000	Dent
N25	5.31	(0.056)	4.72	(0.377)	0.466	0.534	0.000	1.000	0.000	Dent
N6	5.22	(0.067)	6.28	–	0.520	0.480	0.110	0.890	0.000	Dent
SC55	5.48	(0.016)	6.24	(0.244)	0.271	0.729	0.045	0.493	0.462	Dent
SCMALAWI	5.45	(0.108)	6.44	(0.527)	0.263	0.737	0.000	0.609	0.391	Dent
W117U	5.32	(0.027)	5.03	–	0.423	0.577	0.000	1.000	0.000	Dent
A6	5.87	(0.127)	4.46	(0.490)	0.000	1.000	0.000	0.000	1.000	Tropical
L256	5.32	(0.031)	5.63	(0.323)	0.460	0.540	0.465	0.000	0.535	Tropical
BA90	5.41	(0.080)	5.54	(0.139)	0.366	0.634	0.201	0.356	0.443	Tropical
CLA17	5.80	(0.151)	5.67	(0.137)	0.000	1.000	0.000	0.059	0.941	Tropical
CML69	5.64	(0.039)	5.06	(0.416)	0.000	1.000	0.000	0.000	1.000	Tropical
CML245	5.70	(0.133)	5.59	(1.009)	0.330	0.670	0.201	0.273	0.526	Tropical
CML247	5.64	(0.129)	5.12	(0.804)	0.000	1.000	0.000	0.000	1.000	Tropical
CML254	5.50	(0.082)	5.71	(0.814)	0.000	1.000	0.000	0.000	1.000	Tropical
CML287	5.48	(0.042)	5.89	(0.660)	0.000	1.000	0.000	0.000	1.000	Tropical
CML312	5.31	(0.073)	4.06	–	0.000	1.000	0.000	0.000	1.000	Tropical
CML333	5.54	(0.073)	4.88	(0.640)	0.051	0.949	0.023	0.061	0.917	Tropical
CML340	5.51	(0.068)	5.27	–	0.000	1.000	0.000	0.000	1.000	Tropical
CML341	5.50	(0.046)	4.53	–	0.000	1.000	0.000	0.000	1.000	Tropical
CML344	5.58	(0.092)	3.80	–	0.000	1.000	0.000	0.000	1.000	Tropical
CML440	5.60	(0.028)	4.21	–	0.063	0.937	0.063	0.000	0.937	Tropical
CML91	5.44	(0.053)	4.60	(0.802)	0.109	0.891	0.032	0.149	0.819	Tropical
CMLP1	5.60	(0.087)	4.83	(0.020)	0.000	1.000	0.000	0.000	1.000	Tropical
CMLP2	5.59	(0.080)	5.21	(0.457)	0.000	1.000	0.000	0.000	1.000	Tropical
CZL04006	5.51	(0.142)	6.33	–	0.090	0.910	0.000	0.260	0.740	Tropical
CZL0617	5.55	(0.097)	5.27	–	0.000	1.000	0.000	0.000	1.000	Tropical
CZL071	5.30	(0.054)	6.52	–	0.089	0.911	0.028	0.119	0.853	Tropical
EA1197	5.55	(0.124)	5.90	(0.268)	0.234	0.766	0.246	0.000	0.754	Tropical
EA1201	5.56	(0.164)	5.74	(0.492)	0.152	0.848	0.152	0.000	0.848	Tropical
EA1866	5.44	(0.078)	6.47	(0.536)	0.234	0.766	0.237	0.000	0.763	Tropical
EA1712	5.34	(0.012)	6.25	(0.486)	0.199	0.801	0.208	0.000	0.792	Tropical
F2834T	5.44	(0.060)	5.14	(0.431)	0.245	0.755	0.136	0.224	0.640	Tropical
G37	5.65	(0.096)	4.70	(0.010)	0.000	1.000	0.000	0.000	1.000	Tropical
DTPWC9-F115	5.55	(0.072)	5.35	–	0.000	1.000	0.000	0.000	1.000	Tropical
DTPWC9-F104	5.52	(0.030)	4.59	–	0.000	1.000	0.000	0.062	0.938	Tropical
DTPWC9-F31	5.65	(0.068)	4.09	–	0.000	1.000	0.000	0.000	1.000	Tropical
DTPYC9-F74	5.46	(0.092)	5.37	–	0.000	1.000	0.000	0.000	1.000	Tropical
DTPYC9-F46	5.49	(0.105)	5.89	–	0.000	1.000	0.000	0.018	0.982	Tropical
LPSC7-F64	5.45	(0.004)	4.84	–	0.000	1.000	0.000	0.000	1.000	Tropical
LPSC7-F71	5.41	(0.044)	5.49	–	0.000	1.000	0.000	0.000	1.000	Tropical
LPSC7-F103	5.45	(0.019)	4.14	–	0.000	1.000	0.000	0.000	1.000	Tropical
LPSC7-F86	5.49	(0.084)	4.45	–	0.000	1.000	0.000	0.000	1.000	Tropical
H16	5.37	(0.029)	4.36	(0.150)	0.000	1.000	0.000	0.000	1.000	Tropical
KUI44	5.26	(0.101)	4.63	(0.823)	0.050	0.950	0.041	0.016	0.942	Tropical
KUI11	5.54	(0.050)	5.58	(0.073)	0.000	1.000	0.000	0.042	0.958	Tropical
KUI3	5.64	(0.052)	4.17	(0.265)	0.000	1.000	0.000	0.000	1.000	Tropical
LP1037	5.30	(0.037)	6.21	(0.678)	0.340	0.660	0.249	0.175	0.576	Tropical
LP1233	5.39	(0.054)	5.97	(0.458)	0.240	0.760	0.243	0.000	0.757	Tropical
LP35	5.40	(0.158)	5.69	(0.168)	0.243	0.757	0.242	0.008	0.750	Tropical
MO22	5.45	(0.097)	5.40	(0.117)	0.069	0.931	0.065	0.000	0.935	Tropical
NC298	5.75	(0.107)	4.77	(0.815)	0.000	1.000	0.000	0.000	1.000	Tropical
NC304	5.48	(0.026)	5.02	(0.124)	0.000	1.000	0.000	0.000	1.000	Tropical
NC320	5.40	(0.099)	5.61	(0.750)	0.210	0.790	0.000	0.465	0.535	Tropical
NC338	5.78	(0.107)	4.98	(0.145)	0.000	1.000	0.000	0.000	1.000	Tropical
TZI18	5.89	(0.044)	5.39	(0.112)	0.000	1.000	0.000	0.000	1.000	Tropical
ZN6	5.42	(0.036)	5.61	(0.527)	0.249	0.751	0.252	0.000	0.748	Tropical

Genotyping of the 83 inbreds with the Illumina MaizeSNP50 array was either available (Bouchet et al., 2013) or generated for a subset of 11 inbred lines (Data S1). We analyzed 29,090 SNPs contributed by the Panzea project (Zhao et al., 2006) that were developed on a discovery panel of 14 maize and 16 teosinte inbreds. Genotypes of 83 lines on 29,090 SNPs are available in Data S1. We utilized FastStructure v1.0 (Raj, Stephens & Pritchard, 2014) to evaluate the genetic structure of our sample using *K* = 2 and *K* = 3 as the number of genetic groups. We determined the memberships of each inbred to the groups at *K* = 2 and *K* = 3 (Table 1). Kinship was computed from Astle & Balding (2009) using GenABEL (http://www.genabel.org, Aulchenko, Struchalin & Van Duijn, 2010).

Plants from each inbred line were characterized for LER_max_ in the phenotyping facility *Phenodyn* (http://bioweb.supagro.inra.fr/phenodyn/) in two experiments (Data S2). The first experiment included all 83 inbred lines with 3 replicated measurements per inbred. The second experiment was a biological replicate for 58 out of the 83 inbred lines, with 3 replicated measurements. Plants were grown in a Klaszmann substrate (30% clay, 70% peat) according to the protocol reported in Sadok et al. (2007b). Briefly, the LER_max_ (in mm per hour) of the 6th leaf was measured every 15 min during nighttime from 12 to 4 am, time at which LER is maximum. Measurements took place in the 4–7 days during which the leaf elongation rate of leaf 6 has no temporal trend over successive nights (Sadok et al., 2007a). A single measure is therefore an average of LER during 4 to 7 nights. Meristem and air temperature, light intensity and air relative humidity, were measured every 15 min. Plants were grown in the greenhouse with naturally fluctuating conditions (200 to 1,100 µmol m^−2^ s^−1^ at noon time) under well-watered conditions. During the measurement period, meristem temperature was 18.5 °C ± 0.2 °C and 20.0 ± 0.8 °C in Experiment 1 and 2, respectively. Both soil water potential (−0.11 and −0.15 MPa) and vapour pressure deficit (0.93 kPa ± 0.14kPa and 0.98 kPa ± 0.14 kPa) were in the range most favorable for growth during measurements.

In parallel, we measured the GS of 3–5 individuals per inbred line—from the same seed lots used for the LER_max_ measurements (Data S2). Inbreds were grown in a greenhouse in Gif-sur-Yvette (France) and transferred after 3 weeks to the Imagif facility in Gif-sur-Yvette. The total nuclear DNA amount was assessed by flow cytometry according to Marie & Brown (1993) *Pisum sativum* L. ‘Long Express’ (2*C* = 8.37 pg) was used as an internal standard. Leaves of the internal standard and maize lines were chopped using a razor blade in a plastic Petri dish with 1 ml of Gif nuclei-isolation buffer (45 mM MgCl_2_, 30 mM sodium citrate, 60 mM MOPS, 1% (w/v) polyvinylpyrrolidone 10,000, pH 7.2) containing 0.1% (w/v) Triton X–100, supplemented with 5 mM sodium metabisulphite and RNAse (2.5 U/ml). The suspension was filtered through 50 µm nylon mesh. The nuclei were stained with 50 µg/ml propidium iodide and kept 5 min at 4 °C. DNA content of 5,000–10,000 stained nuclei was determined for each sample using a flow cytometer (CyFlow SL3, Partec-Sysmex. Excitation 532 nm, 30 mW; emission through a 630/30 nm band-pass filter). The total 2C DNA value was calculated using the linear relationship between the fluorescent signals from stained nuclei of the maize and the internal standard. We performed three technical replicates per plant. In addition, we employed the inbred line B73 (maize reference genome) to verify the flow cytometer calibration at regular time intervals.

The LER_max_ and GS values were averaged among technical replicates (Data S2). LER_max_ of 58 inbred lines replicated over the two experiments were compared using the Bland and Altman’s method (1986). The replicates were highly concordant with differences between replicates that did not differ from 0 (*t* = − 1.3, *df* = 28, *P* = 0.20), and no correlation between differences between replicates and inbred line mean values (*t* = − 1.6; *df* = 27, *P* = 0.13). GS measurement was replicated on 3–5 plants per line, except for three that were replicated twice and B73 for which we had 14 replicates. Given the high and variable replicates number, the Bland and Altman’s method could not be applied. Instead, we performed a one-way ANOVA and showed that GS variation was mainly owed to inbred line differences (*R*^2^ = 89.7%), with only 10.3% variation across biological replicates. Means and standard deviations for LER_max_ and GS across biological replicates for each inbred line are reported in Table 1, and mean values were used for further statistical analyses. All statistical analyses were performed using the R software.

The effect of genetic groups on LER_max_ and GS was first tested using linear regression on quantitative memberships obtained from FastStructure. We also employed a one-way ANOVA with a qualitative classification of inbreds as Flints, Dents or Tropicals. In this case, inbreds were assigned to a group based on its highest membership coefficient as determined by FastStructure at *K* = 3. We computed pairwise differences between groups using Tukey-Kramer contrasts. We tested the correlation between LER_max_ and GS first by simple regression; second we corrected for genetic structure by adding qualitative or quantitative memberships obtained from FastStructure as covariates in the linear model; third, we used a mixed model declaring FastStructure quantitative membership as a fixed effect and kinship as a random effect (Yu et al., 2006).

## Results

**Figure 1:**
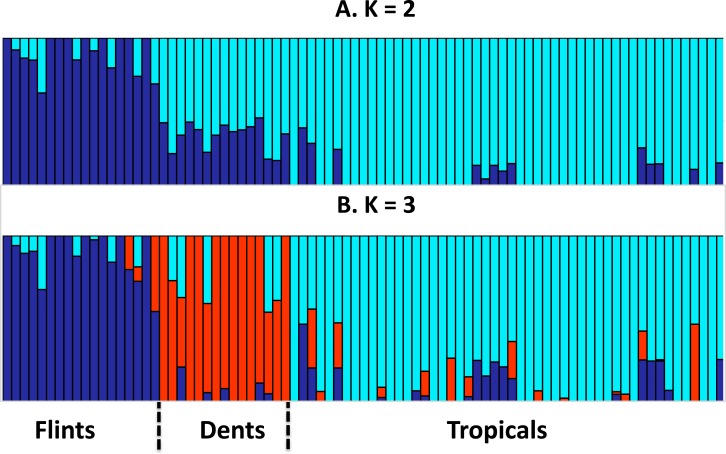
Group membership of 83 maize inbred lines inferred using FastStructure v1.0 (Raj, Stephens & Pritchard, 2014) from 29,090 SNPs with ancestral group number *K* = 2 (A) or *K* = 3 (B). The 83 inbred lines are ordered as in Table 1. Group names were a posteriori defined from the inbred lines with greatest membership with Flints (blue), Dents (red), and Tropicals (cyan).

We assembled a panel of 83 maize inbred lines to test the link between genome size (GS) and the leaf elongation rate (LER_max_). We extracted genotyping data from 29,090 SNPs and assess genetic structuring of the panel. Our results revealed a clear separation between Tropicals and Flints, while Dents were found as admixed individuals when *K* = 2. With *K* = 3, the Dent inbreds form a distinct genetic group (Fig. 1).

**Figure 2:**
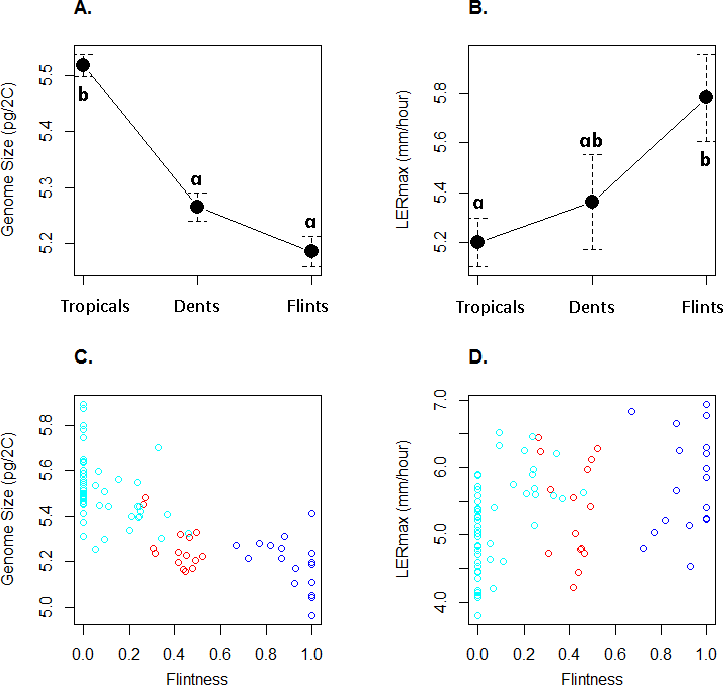
Mean and standard errors across inbred lines for genome size (A) and LERmax (B) for Tropicals, Dents and Flints as defined in Table 1 at *K* = 3. Relationship between genome size (C) and LERmax (D) with Flintness as measured by the membership to the Flint group at *K* = 2 (Table 1). In (A) and (B), pairs of groups with similar letters exhibit non-significant difference in mean values. In (C) and (D), groups are colored as in Fig. 1.

GS varied between 4.96 pg and 5.89 pg (Table 1) with a coefficient of variation of 3.6%. LER_max_ ranged from 3.80 to 6.94 mm h^−1^ (Table 1) with a coefficient of variation of 13.7%. Figure 2 illustrates GS and LER_max_ variation within and among the three genetic groups, each inbred being assigned to the genetic group of greatest membership. For both traits, mean values significantly differed among groups (one-way ANOVA, GS : *F*_(2;80)_ = 52.7, *P* = 2.510^−15^; LER : *F*_(2;80)_ = 4.47, *P* = 0.014). Confirming previous observations, Tropicals displayed a larger genome size than Flints (Chia et al., 2012) while Dents exhibited intermediate GS although non-significantly different from the Flints (Fig. 2A). LER_max_ followed the opposite trend with Flints exhibiting higher values than Tropicals (Fig. 2B). Consistently we found a significant effect of the degree of “Flintness”—membership to the Flint group for *K* = 2—on GS (Fig. 2C) and LER_max_ (Fig. 2D). The Pearson correlation coefficients were highly significant (*r* = − 0.77, *P* = 2.110^−17^ and *r* = 0.40, *P* = 2.010^−4^ respectively for GS and LER_max_).

**Figure 3:**
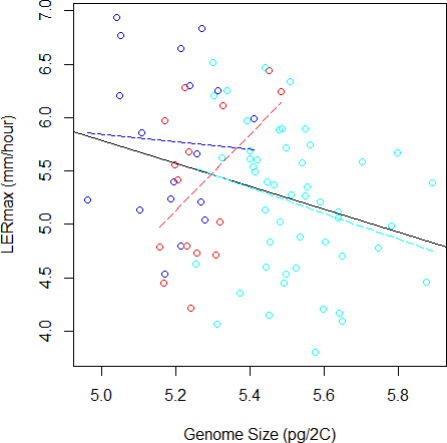
Relation between Genome Size and LERmax within and among groups (Flints in blue, Dents in red, and Tropicals in cyan). The plain line illustrates the linear regression for all data, while colored dotted lines correspond to linear regressions within each group. When the group variable is included in the ANOVA to correct for genetic structure, the relation between LERmax and GS becomes non-significant. The Tropical group, that contains 50 inbred lines, displays a tendency for negative correlation between LERmax and GS.

To validate further this pattern, we investigated the correlation between LER_max_ and GS and found a significantly negative correlation (*r* = − 0.29, *F*_(1;81)_ = 7.28, *P* = 0.008, Fig. 3). However, GS may correlate with relatedness among inbreds because measures of closely related inbreds, i.e., those that form a genetic group, are not independent observations. In order to control this effect, we re-analysed the correlation between GS and LER_max_ controlling for qualitatively (group assignation from the highest membership coefficient) or quantitatively (group membership coefficient) defined groups. We found that the group effect was significant (*F*_(2;77)_ = 4.68, *P* = 0.012). Additionally, the correlation was no longer significant when controlling for either qualitative group origin (*F*_(1;77)_ = 1.07, *P* = 0.31, Fig. 3) or quantitative group membership (*F*_(1;77)_ = 0, 003, *P* = 0.95). As expected when kinship was added to the model, the effect of GS on LER_max_ remained not significant (*P* = 0.95). The regression slope between GS and LER did not differ among groups as indicated by the non-significant Group X GS interaction on the LER measurements (*F*_(2;77)_ = 2.84, *P* = 0.065).

Finally, we performed within-group analyses. Sample size was too limited (15 inbreds) to evaluate correlation within Dents. We found no correlation within Flints (18 inbreds). Tropicals (50 inbreds) however exhibited a negative trend, with small genome inbreds displaying a tendency towards faster growth rate than larger genome inbreds (*r* = − 0.26, *F*_(1;48)_ = 3.35, *P* = 0.073).

## Discussion

That plants with smaller genomes may undergo more rapid replication time of their genome, which translates into faster growth rate than plants with larger genomes, is a prediction of the positive natural selection evolution model of genome size. This prediction is based on findings of positive correlation between GS and duration of the cell cycle in 110 angiosperm species (Francis, Davies & Barlow, 2008). Maize originates from teosintes (Matsuoka et al., 2002) and are characterized by an important range of variation in DNA content (Muñoz Diez et al., 2013). Its genome is extremely fluid (Chia et al., 2012) and GS may evolve rapidly under selection (Rayburn, Dudley & Biradar, 1994). Realini et al. (2015) have recently reported a positive correlation between heterochromatin content and length of the vegetative cycle in 9 maize populations sampled from Northeastern Argentina. However a more direct effect of GS variation on growth rate has never been formally tested.

Here, we determined GS and leaf elongation rate (LER_max_) in 83 improved maize inbred lines selected under contrasted climates. We measured LER_max_ in the developing 6th leaf during the linear phase of elongation, considered as a steady-state (Salah & Tardieu, 1997). This state is commonly used for measuring cell division and/or tissue expansion (Tardieu et al., 2000). It therefore is a good proxy for growth rate in relation with the timing of cell cycle. Besides, the LER_max_ in maize is reproducible and independent of environmental conditions if corrected for temperature effect (Sadok et al., 2007b). It is also a highly heritable trait (Dignat et al., 2013).

Our sample contained inbred lines from three well-defined genetic groups, the Flints, the Dents and the Tropicals. Genetic structuring analysis based on SNP data (Fig. 1) confirmed previous knowledge on inbreds membership to these groups and the recent history of admixture between Tropicals and Flints to form the Dent inbreds at the end of the 19th century (Labate et al., 2003).

Our sample corroborates previous observations from a restricted set of inbreds with temperate inbreds (Flints) exhibiting a significantly smaller GS than tropical (Tropicals) inbreds (Chia et al., 2012) (Figs. 2A and 2C). Interestingly, LER_max_ followed the opposite trend with Flints exhibiting higher values than Tropicals whether inbred group membership was considered as qualitative (Fig. 2B) or a quantitative trait (Fig. 2D). Note that Dents exhibit intermediate values bot for GS and LER_max_ consistent with their admixed status.

At a first glimpse our results therefore support the hypothesis that smaller genomes exhibit a faster development rate. Because LER_max_ is a good indicator of growth ability of other organs including reproductive organs (Dignat et al., 2013), it is tempting to speculate that selection for a faster-life cycle in early flowering Flint inbreds has indirectly impacted genome size.

However the negative correlation between GS and LERmax was mainly driven by among-group variation (Fig. 3), suggesting that the existing link between these variables at the origin of the groups was followed by uncorrelated changes during subsequent evolutionary history. Such a pattern has been reported among species, whereby accounting for the phylogenetic history of species altered the relationship between effective population size and GS (Whitney & Garland , 2010). Noteworthy, within Tropicals smaller genomes displayed a tendency towards faster growth rate than larger genomes. The coefficient of variation of GS was also greater in this group (26%) than in either Flints (22%) or Dents (19%). Tropicals are subjected to high variation in altitude that may exert selective pressure on GS. Additional sampling with limited structuring will be necessary to validate further this result.

Altogether, our results show that selection on GS may have accompanied ancient maize geographical diffusion from its center of origin, consistently with the idea that landraces/inbreds with large DNA content may be excluded from more extreme temperate climates.

##  Supplemental Information

10.7717/peerj.2408/supp-1 Data S1 Genotyping data of 83 inbred lines at 29,090 SNPs

10.7717/peerj.2408/supp-2 Data S2 Leaf elongation rate (LERmax) and Genome size (GS) data

## Additional Information and Declarations

### Data Availability

The following information was supplied regarding data availability: The raw data has been supplied as Supplemental Information.
